# Genetic Variation in SOD1 Gene Promoter Ins/Del and Its Influence on Oxidative Stress in Beta Thalassemia Major Patients

**Published:** 2020-04-01

**Authors:** Poonam Tripathi, Sarita Agarwal, Aditya Narayan Sarangi, Satyendra Tewari, Kausik Mandal

**Affiliations:** 1 Department of Genetics, Sanjay Gandhi Post Graduate Institute of Medical Sciences (SGPGIMS), Lucknow, India; 2 Department of Genetics, Sanjay Gandhi Post Graduate Institute of Medical Sciences (SGPGIMS), Lucknow 226014, Uttar Pradesh, India; 3 Department of Biomedical Informatics Center, Sanjay Gandhi Postgraduate Institute of Medical Sciences, Raebareli Road, Lucknow, India; 4 Department of Cardiology, Sanjay Gandhi Post Graduate Institute of Medical Sciences (SGPGIMS), Lucknow 226014, Uttar Pradesh, India

**Keywords:** Thalassemia, Superoxide dismutase-1, Polymorphism, Hemoglobin

## Abstract

**Background:** A genetic polymorphism of 50 bp insertion/deletion (Ins/Del) (**rs 36232792**) in the promoter region of the SOD1 was reported to influence the enzyme activity. The present study aimed to evaluate the status of this polymorphism of human peripheral blood cells and its association with SOD enzyme activity in beta-thalassemia major patients.

**Material and Methods:** The study was carried out on 200 thalassemia major patients and 200 healthy controls healthy. The SOD1 genotypes were determined using a polymerase chain reaction (PCR)-based method. Serum SOD activity were assessed using SOD assay kit. In-silico analysis was assessed using loss-of-function (LoFtool) (PMID: 27563026).

**Results: **No association was found between the insertion/deletion (Ins/Del) polymorphism and SOD enzyme activity in thalassemia major patients

**Conclusion: **The results of this study indicated that the SOD enzyme activity is not affected by the 50 bp Ins/Del polymorphism of SOD1in thalassemia major patients. Further research with larger sample size and with other genes of antioxidant system is required.

## Introduction

 Beta-thalassemia major is the most common hereditary anemia resulting from homozygosity or compound heterozygosity to beta-globin (HBB) gene mutations with severe reduction or total absence of beta globin chains. A higher frequency of this disease was found in the Mediterranean region, Africa, South-East Asia and the Indian subcontinent ^1^^, ^^2^. The prevalence of thalassemia major is about 3% all around the world and World Health Organization (WHO) estimates that at least 6.5% of the world populations are carrier of different inherited disorders of hemoglobin^6^.

These patients are severely anemic and lifelong blood transfusions are required for survival. Regular blood transfusions lead to iron overload and require iron-chelation therapy. 

Transition metals such as iron play important role in the generation of reactive oxygen species (ROS) and free radicals, which are supposed to damage cellular and subcellular structures and cause metabolic dysfunction. The disturbed iron metabolism generates oxygen-derived free radicals in thalassemias. This condition, together with impaired natural factors and mechanisms involved in detoxification of ROS and free radicals, results in extensive oxidative stress ^1^^, ^^2^^, ^^5^^.^ This oxidative stress and possible consequential events accelerated apoptosis that may contribute to shortened life span of erythrocytes. Thus, level of oxidative stress and pro-inflammation factors are required to be monitored in these patients.

Different enzymes involved in defense mechanism against ROS play an important role in regulating proper antioxidant levels in humans. SOD1 is one of the three human superoxide dismutase (antioxidant enzyme) identified and characterized in mammals: copper-zinc superoxide dismutase (Cu/ZnSOD1), manganese superoxide dismutase (MnSOD or SOD2), and extracellular superoxide dismutase (ECSOD or SOD3)^4^. 

The human SOD1 gene (Entrez Gene ID 6647) is located on chromosome 21q22.11. More precisely, this gene is located from base pair 33,031,935 to base pair 33,041,241 with a genomic size of 9307 bp according to UCSC Genome Browser (GRCh37/ hg19; http://genome.ucsc.edu/)^3^. The Sod1 gene consists of five exons interrupted by four introns. A number of polymorphisms have been identified in SOD1 gene, mainly affects regulatory regions of gene, including promoter, UTRs, and introns^16^. A 50 bp deletion polymorphism (**rs 36232792**) in SOD1 promoter region (1684 bp upstream of the ATG start codon) has been recognized. In-vitro analyses have demonstrated that the SOD1 50 bp deletion is associated with decreased promoter activity and low mRNA levels in cells, which is caused by the loss of two Sp1 binding sites ^21^. 

Several studies focused on the genetic and biochemical characterization of SOD1, demonstrating that SOD1 plays an important role in diseases as heart failure^15^, cancer ^16^, diabetes^17^,Down’s syndrome ^18^, and amyotrophic lateral sclerosis ^19^. 

The 50 bp-deleted region which binds to multiple transcription factors has been shown to be associated with reduced promoter and enzymatic activity of SOD1 ^24^^, ^^25^^, ^^28^. Several studies in which associations of SOD1 gene variants with several diseases like type 2 diabetes^22^^,^^13^, cardiovascular related risk factors^23^, temperament and heroin dependency^28^^,^^29^ have been reported; however, there are no study investigating association of SOD1 50-bp deletion variant with oxidative injury in thalassemia major patients. Therefore, the present study aimed to find out the status of 50-bp Insertion/deletion polymorphism of SOD1 gene promoter in thalassemia major cases and normal healthy individuals. However, the SOD enzyme activity was measured in thalassemia major patients only and compared among genotypes observed within that group. 

## MATERIALS AND METHODS

**Study Population and Sample collection**

In the present study, patients affected by homozygous beta-thalassemia, 61 females and 139 males, above 10 years, were recruited after obtaining written informed consent. This study was approved by the Bioethics Committee of the institute Lucknow. The study included 200 thalassemia major patients and 200 healthy controls. All patients had been previously characterized for β-globin gene mutation, received blood transfusion to maintain hemoglobin levels above 9.5 g/dL and were under iron chelation therapy with Deferoxamine (DFO).

We collected 2 ml of venous blood in EDTA and 2 ml in plain tubes separately from patients and normal healthy controls. Blood from each subject was assayed for serum concentration of ferritin and SOD enzymatic Assay. Genomic DNA was extracted from peripheral blood by phenol-chloroform method described by Poncz et al. [1983]. The quantification of the DNA was measured on spectrophotometer at wavelength of 260 nm and the quality was checked on 0.8% agarose gel. 

**Identification of the SOD1 50 bp Ins/Del polymorphism**

**Figure 1:**
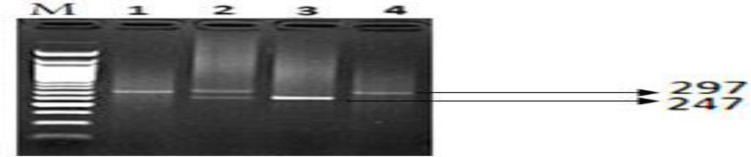
Gel picture of the PCR products of the 50 bp Ins/Del of SOD1 gene on 2% agarose gel. Lane 1, 4: Ins/Ins; Lanes 2: Ins/Del; Lane 3: del/del; M: 50 bp DNA marker

The polymorphic (rs 36232792) site at SOD1 promoter (1684 bp upstream of the ATG start codon) was amplified by polymerase chain reaction (PCR) using forward primer: AATTCCTTACCCCTGTTCTA and reverse primer: GGCAGATTTCAGTTCATTGT. The PCR condition were denaturation at 95°C for 5 min, followed by 30 cycles at 95°C for 30 secs, 62°C for 30 secs, 72°C for 25 secs, and then by a final extension at 72°C for 10 min^3^. The resultant polymerase chain reaction (PCR) products yielded 2 DNA fragments of 297 and 247 bp for the Ins and Del alleles, respectively, on 2% agarose gel as presented in Figure 1.

**Figure 2:**
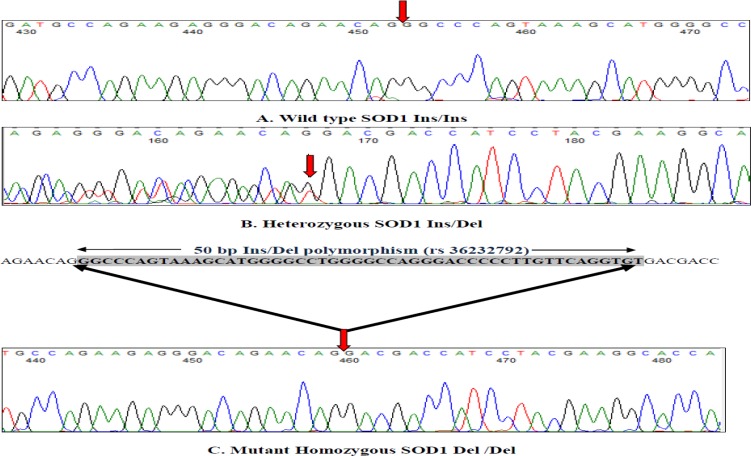
Sequencing results of SOD1 gene promoter 50 bp Ins/Del polymorphism [rs 36232792] (A-wild type Ins/Ins, B- Heterozygous Ins/Del, C- Mutant homozygous Del/Del) red arrow indicates the Insertion and deletion site in SOD1 gene promoter.

Sequencing of PCR products was performed by using the BigDye Terminator v3.1 Cycle Sequencing Kit (Applied Biosystems, Foster City, CA, USA), and products were resolved on the ABI 3130XL Genetic Analyzer (Applied Biosystems). Sequence electropherograms were analyzed by using Finch TV (Figure 2). 

**SOD activity measurement**

Serum SOD activity was assessed using SOD assay kit (Cayman, Superoxide dismutase Assay Kit, Item No.706002) based on utilization of tetrazolium salt for detection of superoxide radicals generated by xanthine oxidase and hypoxanthine. The dynamic range of the kit was 0.005-0.050 units/ml SOD. The absorbance of the samples was measured by using a microplate reader at 450 nm.

**In-silico analysis**

Significance of the upstream non-coding indel (rs36232792) was assessed using loss-of-function (LoFtool) (PMID: 27563026), which provides a percentile gene intolerance score to functional change, based on loss-of-function variants and tissue expression in a large group of 60706 unrelated individuals. The lower the score, the higher gene intolerance to functional change.

**Statistical analysis**

All statistical analyses were performed using the SPSS software for Windows, version 16.0 (SPSS Inc, Chicago IL, USA). Comparisons between quantitative variables were carried out after data explored for normality using Kolmogorov-Smirnov test of normality. Statistical difference and genetic comparisons between groups were evaluated by Student-t test, Chi square tests and ANOVA tests with 95% confidence interval when appropriate for quantitative variables. Logistic regression was used for computation of ORs and 95% CIs (Confidence Interval). The linear and binary logistic regression analyses were performed to evaluate the effect of different genotypic variation on SOD activity.

The demographic and clinical data were expressed as mean ± standard deviation (SD) for continuous variables and percentages for discrete variables and p values were calculated using, Chi-square test, Students’ paired *t*-test or Manne Whitney U test. The correlation coefficient (r) which is a measure of the degree of closeness of the linear relationship between two variables was determined. Univariate spearman’s correlation test was performed to identify the correlation between genotype and different parameters.

## Results

 In the present study, allele and genotype frequencies of 50 bp Ins/Del polymorphisms (**rs 36232792)** in SOD1gene promoter were compared between 200 thalassemia cases and 200 controls (Table 1). 

**Table 1:** Distribution of Genotype frequency between Beta Thalassemia major patients and control population

**SOD1 ** **Genotype**	**Patients** **(n=200)**	**Control** **(n=200)**	**OR** **(95%CI)**	**P**	**Risk ** **ratio**
Ins/Ins	155 (77.5%)	184 (92%)	-	Ref	-
Ins/Del	43 (21.5%)	16 (16%)	3.1903 (1.729 to 5.886)	**0.0002**	2.00
Del/Del*	2 (2%)	0 (0%)	-	-	-
Allele Frequency					
Ins	353 (88.3%)	384 (96%)	-	Ref	-
Del	47 (11.7%)	16 (4%)	3.1955 (1.779 to 5.738)	**0.0001**	2.05

P was calculated by Chi-square test with (2 x 2) contingency

Table and considered <.0.05 as significant.

Abbreviations: SOD, Superoxide dismutase; CI, confidence interval; OR, odds ratio.

* (For Del/Del genotype the number obtained was not sufficient to calculate OR and P)

The genotype frequencies of Ins/del genotypes between thalassemia patients and control group were statistically significant (Table 1, P = 0.0002 for Ins/Del, and for Del/Del P value was not calculated due less sample size). In addition, there were no significant differences between the patients (2%) and control groups (0%) for Del/Del variant. While, calculating the allelic distribution Del allele ( SOD1 50bp Ins/ Del) was found to be more prevalent in the thalassemia patients with the frequency of 11.7% and 4% in cases and controls, respectively, and also statistically significant (OR=3.1955, 95% CI: 1.779-5.738, *P*= 0.0001). Although the frequency of the Del/Del variant did not differ significantly between the study and control groups , patients with Del allele were at a 3.2-fold greater risk of oxidative stress than those with the Ins variant [odds ratio: 3.1955 (95% CI: 1.779-5.738)] (Table -1). Demographic information and biochemical parameters of the patients are summarized in Table-2. 

**Table 2:** Demographic and Clinical Characteristic of thalassemia major patients

**Parameters**	**Mean±SD**
Age	16.36±5.08 years
Gender Male Female	139 (69.5%) 61 (30.5%)
Serum Ferritin	3457.80±1114.61 ng/mL

**Figure 3:**
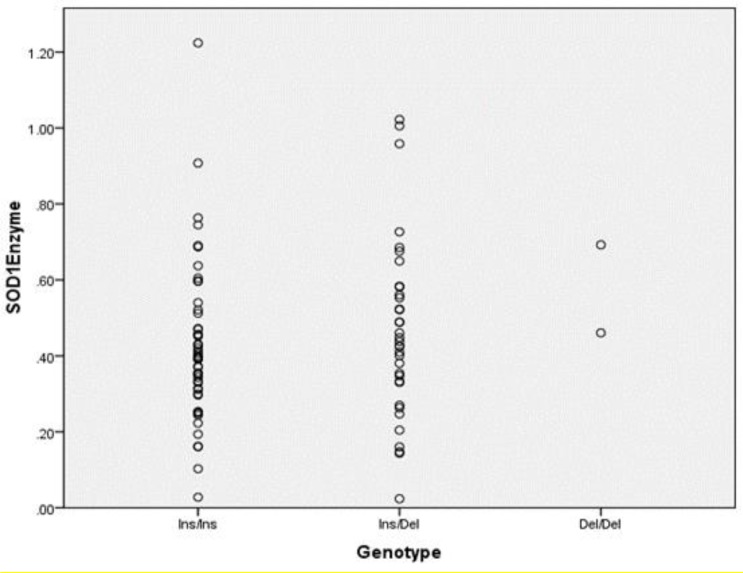
SOD enzyme activity according to different genotypes observed

Since in this study only a fraction of thalassemia patients took SOD1 enzyme activity, when compared with in genotype, their serum SOD activity was not statistically significantly vary between different genotypes in patients (*P*>0.0001) (Table 3) (Figure 3). No significant correlation existed between various genotypes, age and ferritin levels of the patients examined throughout this study.

**Table 3:** Mean serum superoxide dismutase (SOD1) activity in relation to SOD1 gene 50 bp Ins/Del polymorphism.

**Genotype**	**SOD 1 Enzyme Activity** **Mean±SD**
Ins/Ins	0.43 ± 0.20
Ins/Del	0.46 ± 0.23
Del/Del	0.57± 0.16

Silico analysis performed by using Loss of function (Lof) Tool Score for the variation rs36232792 was 0.126 and the variation was predicted to have probably damaging effect.

## Discussion

 The antioxidant enzymes play an important role in scavenging ROS produced under oxidative stress (Ramchandra*et al*. 2012). Moreover, previous studies examining the association between ROS and various diseases have revealed that excessive oxidative stress or decreased antioxidant activity can cause several pathologic states. Defects in antioxidant pathways are connected to several different types of diseases, including diabetes, age-related diseases and cancer^8^^, ^^9^^, ^^10^. Any genetic variations in the SOD genes coding for the SOD enzymes may lead to decreased or impaired regulation of their enzymatic activity and alter ROS detoxification^12^. Among the antioxidant enzymes, SOD is considered to be important in the oxidant defense mechanism, as it is involved in the first line of defense and detoxify superoxide ions^11^. 

Our findings in SOD1 gene promoter for 50 bp Ins/Del polymorphic variation (**rs 36232792)** revealed the serum SOD activity in thalassemia patients, but did not reach statistical significance. Also, it was revealed from the present study that Ins/Del genotypes were not associated with decreased enzyme activity in patients; however, on calculating allele frequency, P value was found to be statistically significant in thalassemia major patients. In silico analysis, the results provide only predictions for the variation (**rs 36232792) **that has probably damaging effect, but these results require further confirmation using methods such as functional studies in animal models.

SOD1 accounts for ~85% of the total cellular SOD activity of most mammalian cells and is highly active in the human kidney and in the vascular wall^4^. The SOD1 promoter region is of great importance for the regulation of SOD1 mRNA levels as it harbors the binding sites for several transcription factors^8^ and sequence differences in these cis-acting responsive elements may be causative for varied mRNA expression. In vitro analysis demonstrated that the 50 bp deletion polymorphism in SOD1 promoter (1684 bp upstream of the ATG start codon) is associated with reduced promoter activity and deficient SOD1 expression in cells because of the loss of two Sp1 (specificity protein 1) binding sites^4^. SOD1 deficiency is believed to result in increased levels of vascular superoxide ^24^, hypertrophy of arteries and peroxynitrite and impaired endothelium-dependent relaxation in large arteries and microvessels^24^^, ^^25^^, ^^26^.

Data prepared from the several studies emphasize that antioxidant enzyme gene polymorphisms and antioxidant enzymes became the area of interest as pharmacological targets to reduce ROS production, and provide a strategy to prevent or slow the progression of oxidative injury in patients.

In the present study, we investigated whether genetic polymorphisms in SOD1 contribute to the risk of developing oxidative stress in thalassemia patients in hospital based case–control study. We observed no statistically significant risk of oxidative stress associated with the (**rs 36232792**) 50 bp Ins/Del polymorphism. Although in the combined analysis of the Ins and Del alleles of SOD1 gene promoter variation the Del alleles were found to be statistically significantly related to increased risk of oxidative stress in patients than control, further extended studies with large sample size is required for confirmation and validation of present findings. 

## CONCLUSION 

 The oxidative stress (caused by ROS) plays a central role in the pathogenesis of anemia in beta-thalassemia. Nowadays, there has been much interest and research on single nucleotide substitutions (SNPs) in order to understand the maintenance of such polymorphisms in human populations (Cassia *et al*, 2011). Moreover, several SNPs have been also reported to result in changes of the levels or the activities of antioxidant enzymes, which can lead to reduction in protection against oxidative stress. 

Here, we determine the status of (**rs 36232792)** 50 bp Ins/Del SOD1 gene promoter polymorphism in thalassemia major patients and its effect on enzyme activity, but no statistically significant association was observed. However, interestingly Del alleles were found to be significantly related to increased risk in patients than control. So, future studies should be designed to evaluate in vivo novel antioxidant strategies with multitarget effects on erythropoiesis with the final goal to know the status of anemia in beta-thalassemia major patients. Further research with larger sample size and with other genes of antioxidant system is recommended in relation to different complications in thalassemia major patients.
